# Photostable and Small YVO_4_:Yb,Er Upconversion Nanoparticles in Water

**DOI:** 10.3390/nano11061535

**Published:** 2021-06-10

**Authors:** Masfer Alkahtani, Anfal Alfahd, Najla Alsofyani, Anas A. Almuqhim, Hussam Qassem, Abdullah A. Alshehri, Fahad A. Almughem, Philip Hemmer

**Affiliations:** 1 National Center for Renewable Energy, King Abdulaziz City for Science and Technology (KACST), Riyadh 11442, Saudi Arabia; aalfahd@kacst.edu.sa (A.A.); nalsofyani@kacst.edu.sa (N.A.); amukhem@kacst.edu.sa (A.A.A.); hqasem@kacst.edu.sa (H.Q.); 2 Institute for Quantum Science and Engineering, Texas A&M University, College Station, TX 77843, USA; prhemmer@exchange.tamu.edu; 3 National Center for Pharmaceutical Technology, King Abdulaziz City for Science and Technology (KACST), Riyadh 11442, Saudi Arabia; abdualshehri@kacst.edu.sa (A.A.A.); falmughem@kacst.edu.sa (F.A.A.); 4 Department of Electrical and Computer Engineering, Texas A&M University, College Station, TX 77843, USA; 5 Zavoisky Physical-Technical Institute, Federal Research Center “Kazan Scientific Center of RAS”, 420029 Kazan, Russia

**Keywords:** upconversion nanoparticles, silica-coated UCNPs, bio-imaging, bio-application

## Abstract

In this work, we report a simple method of silica coating of upconversion nanoparticles (UCNPs) to obtain well-crystalline particles that remain small and not agglomerated after high-temperature post-annealing, and produce bright visible emission when pumped with near-infrared light. This enables many interesting biological applications, including high-contrast and deep tissue imaging, quantum sensing and super-resolution microscopy. These VO_4_-based UNCPs are an attractive alternative to fluoride-based crystals for water-based biosensing applications.

## 1. Introduction

Lanthanide-doped upconversion nanoparticles (UCNPs) absorb one or more low-energy NIR photons to emit one high-energy visible photon [1,2,3,4]. This property greatly suppresses the biofluorescence background that limits other fluorescent probes. As a result, interest in UCNPs is rapidly growing for numerous biological applications, such as in background-free imaging, quantum sensing, drug delivery, and super-resolution microscopy [5,6,7]. Compared to upconversion dyes and quantum dots (QDS), UCNPs achieve efficient upconversion at low pump laser intensities, comparable to single-photon down-converting systems. This is because they have real long-lived metastable states with lifetimes on the microsecond scale. As a result, UCNPs provide photostable and tunable photoluminescence when excited within the tissue optical transparency window by inexpensive continuous-wave NIR lasers [8,9,10,11,12,13]. Furthermore, UCNPs have shown minimal toxicity to biological tissues, which makes them one of the safest fluorescent probes [11,14,15,16,17].

Lanthanide ions doped in fluoride crystals are known as the best and most efficient UCNPs in many interesting applications, with strong up-conversion luminescence (UCL) [8,9]. This is because fluoride crystals have a relatively low phonon energy so that the intermediate state can only decay by highly multi-phonon relaxations [18,19,20]. They work efficiently in organic solvents such as cyclohexane, but their UCL was reported to drop significantly when dispersed in water [21]. This is due to quenching of the intermediate state by water in contact with the surface of the UCNP, also to defects inherent to their low synthesis temperature [8]. To overcome these limitations, the use of core/shell or core/multi-shell strategies [8,9,11], and hydrophilic surface modification protocols [22], have been used. While successful, these significantly complicate their chemical synthesis.

So far, the best alternative to fluoride-based UCNPs is lanthanide ions doped in oxide crystals such as YVO_4_ nanoparticles, which have shown a good upconversion efficiency in water, even for small particles without any surface modifications [18,23,24]. In general, oxide nanoparticles are obtained by colloidal synthesis at lower temperatures than fluoride-based UCNPs, and this gives them poor overall crystallinity and defect-enhanced surface quenching effects, which greatly reduces their UCL efficiency [23,24,25]. Post-annealing at high temperature is an effective solution to this problem [18,24,25]. However, the post-annealing process leads to unequal particle sizes and agglomerations. Many studies have reported that this challenge can be overcome by silica encapsulation during high temperature annealing, which allows the recovery of good crystalline properties without an excessive size increase [18,25]. However, most of these studies start with relatively large particles (50 nm average size) instead of the ultra-small and well-dispersed UCNPs that are desirable for many biosensing applications [18,23,25,26,27].

In this work, we report an easy and efficient method to synthesize a small (10 nm) VO_4_-based UCNP, based on citrate size capping followed by a silica encapsulation for a protected calcination process. The recovered UCNPs retained their original small size with good up-conversion efficiency. The resulting UCNP particles are highly water dispersible, as desired for in vivo imaging.

## 2. Materials and Methods

### 2.1. Synthesis of YVO_4_: Er^+3^, Yb^+3^ Nanoparticles

Raw materials for this experiment, Y(NO_3_)_3_ 4H_2_O, Yb(NO_3_)_3_ 5H_2_O, Er(NO_3_)_3_ 5H_2_O, sodium citrate, sodium orthovanadate (Na_3_VO_4_), and tetraethylorthosilicate (TEOS 98%) were purchased from Sigma Aldrich (St. Louis, MO, USA). In a water bath at 60 °C, a solid mixture of Y(NO_3_)_3_ 4H_2_O (0.270 g, 7.78 × 10^−4^ mol), Yb(NO_3_)_3_ 5H_2_O (0.089 g, 1.99 × 10^−4^ mol) and Er(NO_3_)_3_ 5H_2_O (0.0089 g, 2.0 × 10^−5^ mol) was dissolved in 10 mL of deionized water. The resulting solution of Ln(NO_3_)_3_ was added to an aqueous solution of (0.1 M) sodium citrate (0.22 g in 7.5 mL of deionized water) under vigorous stirring. During the addition, a white precipitate of lanthanide citrate was immediately formed. An aqueous solution of sodium orthovanadate (Na_3_VO_4_) (0.1 M, pH = 12) was prepared by dissolving 0.1839 g of Na_3_VO_4_ in 10 mL of deionized water and then added dropwise to the above mixture until the precipitate was completely dissolved. After constant stirring for 1 h at 60 °C, the final YVO_4_:YbEr nanoparticles were obtained in a clear solution, with a pH of 8.0.

### 2.2. Synthesis of YVO_4_: Er^+3^, Yb^+3^@SiO_2_ Core-Shell Structured Nanoparticles

YVO_4_: Er^+3^, Yb^+3^ (10 mL) was added to 40 mL of ethanol and ultrasonicated for 30 min. Afterwards, 1 mL of ammonia (full concentration) plus 0.15 mL of tetraethylorthosilicate (TEOS 98%) were added to above solution and stirred at room temperature for 4 h. The resulting nanoparticles coated with silica were collected by centrifugation at 6000 rpm for 10 min, washed three times with ethanol/water (1:1 *v*/*v*), and then dried at 60 °C overnight. The white powder was then calcinated at 700–750 °C for 2 h.

### 2.3. Silica Shell Removal by NaOH Solution

The calcinated particles were dispersed in aqueous NaOH solution (10 mL, 0.5 M) under stirring for 4 h to remove the silica matrix from particles. The nanoparticles were collected by centrifugation and washed three times with deionized water. The final product was dispersed in deionized water.

### 2.4. In Vitro Cytotoxicity Assays for Synthesized UCNPs

The investigation of nanoparticles interactions with living tissues is an essential way to evaluate the toxicity, in vitro. The biotoxicity of the UCNPs was performed against two living cellular models supplied from the American Type Culture Collection (ATCC, Manassas, VA, USA), which are mouse skin cancer cell line (B16-F10, ATCC number: CRL-6475) and human lung carcinoma cell line (A549, ATCC number: CCL-185). Both cells were used between passages 7 and 20, and regularly maintained in complete cell culture growth medium composed of Dulbecco’s modified Eagle’s medium (DMEM), 1% (*v*/*v*) antibiotic solution (penicillin–streptomycin combination) and 10% (*v*/*v*) fetal bovine serum (FBS).

In vitro cytotoxicity of the UCNPs was evaluated using two colorimetric assays to quantify UCNPs effect on the cellular membrane integrity and the cellular metabolic activity of B16-F10 and A549 cells. Lactate dehydrogenase (LDH) enzyme is a cytoplasmic enzyme that can be released and quantified in cell culture media when the treated cellular membrane is being disrupted using LDH assay kit (Sigma-Aldrich, Poole, UK). The cell viability can be detected also using 3-(4,5-dimethylthiazol2-yl)-5-(3-carboxymethoxyphenyl)-2-(4-sulfophenyl)-2H-tetrazolium (MTS)assay. The MTS assay was used to evaluate the cellular metabolic activity of the cell lines using MTS cell proliferation reagent (Promega, Southampton, UK). In a 96-well plate, B16-F10 and A549 cells were initially counted and seeded (100 µL/well, seeding density of 1 × 10^4^ cells/well), and then incubated overnight at 37 °C. Increasing doses of the UCNPs (from 0.39 to 800 µg/mL) were then applied to B16-F10 and A549 cells and incubated at 37 °C for 4 h in the presence of complete cell culture growth medium. In nanoparticle-free wells, 0.2% (*v*/*v*) Triton X-100 lysis solution and DMEM were applied to cells as a positive and a negative cytotoxicity control, respectively.

Following the incubation of the UCNPs with both cells, the LDH assay was conducted in a new 96-well plate to transfer 50 μL of the treated samples containing DMEM and the cytoplasmic LDH enzyme, which are being released from the disrupted cellular membrane. Next, 100 μL of the LDH reaction mixture was then pipetted into each well and incubated at room temperature for 30 min. Next, the absorbance at 490 nm was measured using the microplate reader (Cytation™ 3, BioTek Instruments Inc., Winooski, VT, USA). The percentage of LDH release and cell viability were calculated using the following Equations:

*LDH release* (%) = (*S* − *H*) / (*T* − *H*) × 100 (1)

*Cell viability* (%) = 100 − *LDH release* (%) (2)

where *S* is the absorbance of the cells treated with the UCNPs, *H* is the absorbance of the negative cytotoxicity control, and *T* is the absorbance of the positive cytotoxicity control.

For the MTS assay, the samples were removed from the wells and 100 μL of fresh complete cell culture medium mixed with 20 μL of the MTS solution was then pipetted into each well. The plates were then incubated at 37 °C for a further 3 h. Cytation™ 3 microplate reader was then used to measure the MTS absorbance at 490 nm. The percentage of cell viability was calculated using the following equation:

*Cell viability* (%) = (*S* − *T*) / (*H* − *T*) × 100 (3)

## 3. Results and Discussion

Experimentally, YVO_4_:Yb,Er UCNPs were synthesized following a modified sodium citrate synthesis protocol reported in [28]. The sodium citrate protocol was chosen as the citrate complexing agent, which are known to limit the size of particles during growth to less than 10 nm, and also to increase their stability. Briefly, an appropriate amount of lanthanide ions salt was dissolved into deionized water (DI) and mixed with sodium citrate to form a lanthanide citrate mixture (see Material and Methods section for details). Then, an aqueous solution of sodium orthovanadate salt was added dropwise to the lanthanide citrate mixture to form ultrasmall YVO_4_:Yb,Er UCNPs, after constant stirring for one hour at 60 °C. The synthesized UCNPs showed an average size less than 10 nm, but with poor optical and crystal structure properties. Therefore, protected high-temperature annealing is required.

**Figure 1:**
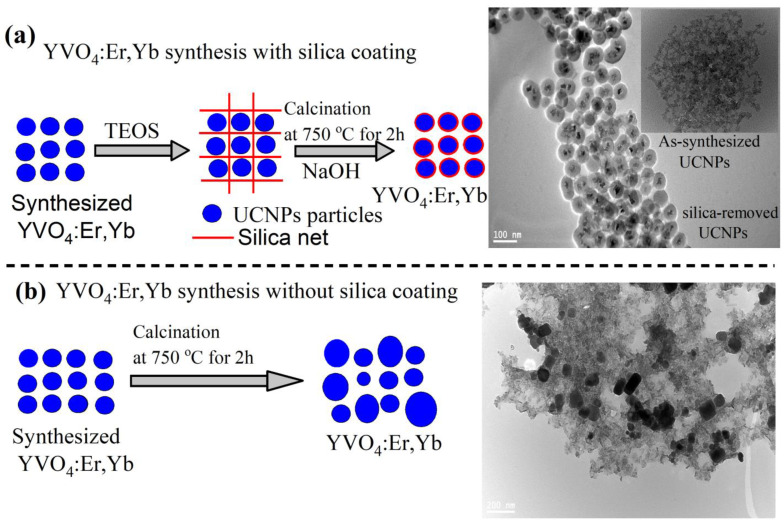
An illustration of high-temperature calcination of YVO_4_:Yb,Er UCNPs. (**a**) Protected calcination of UCNPs by silica coating resulting in well size-controlled and dispersed UCNPs particles. (**b**) Direct calcination process of the UCNPs to 750 °C for 2 h without silica coating, which results in a large size distribution and agglomerated UCNPs.

**Figure 2:**
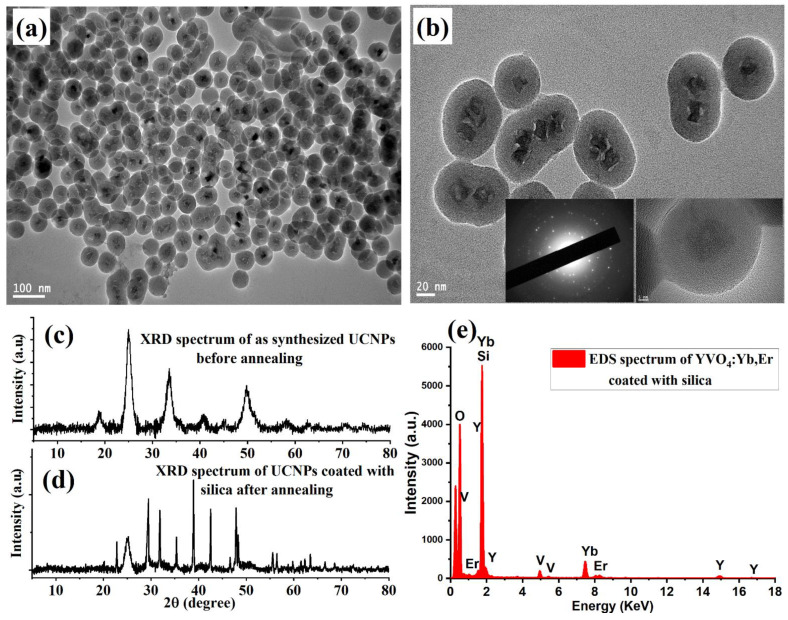
Structural analysis and characterization of YVO_4_:Yb, Er UCNPs. (**a**) Low magnification TEM image of well-crystalline and small size UCNPs obtained by the protected calcination process. (**b**) High magnification of the synthesized UCNPs. (**c**,**d**) XRD data of UCNPs before and after high-temperature treatment. (**e**) Energy-dispersive X-ray (EDX) spectrum recorded and analyzed from the synthesized UCNPs.

For this purpose, we designed two experiments with and without silica coating, as illustrated in Figure 1a,b. In sample 1, illustrated in Figure 1a, the prepared particles were coated with silica in a core/shell structure following a silica-coating procedure reported in [25] and detailed in the Material and Methods section. The silica-coated UCNPs were then annealed to 750 °C for 2 h in air, using a high-temperature tube furnace. The silica coating was then partially removed by an NaOH solution incubation treatment to obtain dispersed and highly crystalline YVO_4_:Yb,Er nanoparticles, as illustrated in a the transmission electron microscope (TEM) image shown in Figure 1a and Figure 2. The final silica-coating thickness can be controlled by either the concentration of the NaOH solution or the incubation time as suggested in [25]. To appreciate the important role of silica encapsulation during high-temperature annealing, the prepared UCNPs in sample 2 were directly annealed to 750 °C for 2 h without silica coating. As a result, a large size distribution and significant agglomeration was seen in the TEM image in Figure 1b. Because sample 2 was introduced for comparison only, it will not be investigated any further.

The shape, size, and elemental composition of the synthesized silica-coated UCNP particles were characterized using a JEM-2100F TEM microscope (JEOL, Peabody, MA, USA) operating at an acceleration voltage of 200 kV TEM microscope. For this, a 1 µL sample was dropped onto carbon TEM grids for imaging. Low- and high-magnification TEM images showed dispersed and well-crystalline UCNP particles, with average sizes of 10–15 nm, as demonstrated in Figure 2a,b. The crystallinity of the synthesized YVO_4_:Yb,Er nanoparticles before and after the protected calcination treatment was investigated using X-ray diffraction (XRD, Malvern, Westborough, MA, USA). Figure 2c shows weak and broad XRD intensity peaks of the synthesized UCNPs before high-temperature annealing treatment, due to the poor overall crystallinity. After protected high-temperature annealing at 750 °C, the XRD patterns revealed strong and sharp peak intensity after calcination, as illustrated in Figure 2d. The narrow XRD peaks indicate well-crystalline particles, which can result in good optical properties. The energy-dispersive X-ray (EDX) spectrum revealed the expected composition elements of the synthesized UCNPs, as illustrated in Figure 2e.

**Figure 3:**
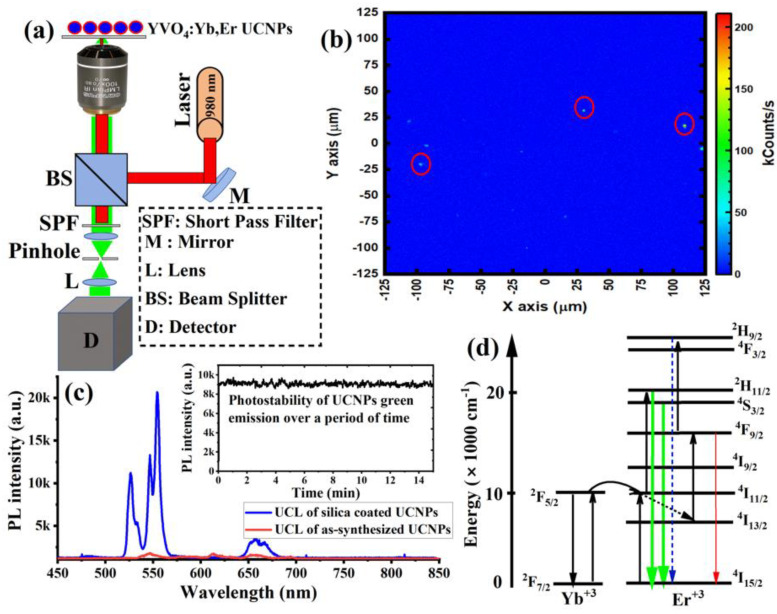
(**a**) An illustration of optical setup used for optical characterizations in this study. The optical setup consists of 980 nm excitation laser, custom-built confocal microscope, and spectrometer. (**b**) Optical scan of the spin-coated UCNPs under 980 nm laser illumination. (**c**) Upconversion luminescence (UCL) spectrum of the synthesized UCNPs before and after high-temperature treatment. UCL of synthesized UCNPs showed a very weak spectrum due to overall poor crystallinity. The fluorescence of the UCNPs after high-temperature treatment showed two strong green emissions at 525 nm and 550 nm, and a weak emission at 650 nm. (**d**) Electronic structure and upconversion energy transfer processes between energy synthesizer (Yb^+3^) and activator ion (Er^+3^) under 980 nm laser excitation (photon upconversion).

Next, to study the optical properties of the synthesized UCNPs, 1 µL of the sample was spin-coated on a quartz slide to make a thin layer of the sample for optical characterizations. The spin-coating technique was used to avoid agglomeration during drying. The UCNPs sample was then placed on a custom-made confocal laser-scanning microscope equipped with a continuous wave (CW) 980 nm NIR laser excitation, optical spectrometer, and a single-photon counter, as illustrated in Figure 3a. Figure 3b shows the optical scan of the spin-coated UCNPs under a 980 nm laser at an illumination intensity of 8 kW/cm^2^. The optical spectrum from each spot in the image revealed a clear UCL spectrum, with two strong green emissions peaked at 520 nm and 550 nm, and a weak red emission peaked at 650 nm, as shown in Figure 3c. The photostability of the UCL green emission, which peaked at 525 and 550 nm from individual UCNP particles (after high-temperature treatment) in the optical scan, was evaluated over 15 min. Figure 3c (inset) showed neither photobleaching nor blinking from the synthesized UCNPs under continuous NIR excitation. In contrast, synthesized UCNPs (before high-temperature treatment) showed a very weak UCL spectrum, as shown in Figure 3c.

Figure 3d explains the UCL optical process in UCNPs. In this optical process, initially, Yb^+3^ ions absorb the first NIR photon of the 980 nm laser to populate the (^2^*F*_5/2_) *Yb*^+3^ excited state. After that, energy transfer from this state promotes *Er*^+3^ ions to its quasi-resonant metastable state ^4^*I*_11/2_. Due to a two-photon optical process, a second NIR photon absorption re- excites the *Yb*^+3^ back to the (2*F*_5/2_) *Yb*^+3^ excited state, and energy transfer further excites the *Er*^+3^ (^4^*I*_11/2_) metastable state to a higher excited (^4^*F*_7/2_) *Er*^+3^ state. After the two-photon excitation is complete, the highly excited state (^4^*F*_7/2_) of Er^+3^ then relaxes to (^2^*H*_11/2_, ^4^*S*_3/2_, and ^4^*F*_9/2_) *Er*^+3^ states through multi-phonon relaxations. Consequently, radiative transitions then occur to the ground state ^4^*I*_15/2_ of the *Er*^+3^, according to the following transitions: ^2^*H*_11/2_ → ^4^*I*_15/2_, ^4^*S*_3/2_ → ^4^*I*_15/2_, and ^4^*F*_9/2_ → ^4^*I*_15/2_, which give green emission peaks at 525 nm and 550 nm, and red emission peaks at 650 nm.

As biocompatibility is the most important criteria for successful fluorescent markers, we performed an in vitro toxicity evaluation of the synthesized UCNPs to confirm the safety and the biocompatibility of the applied nanoparticles to biomedical applications. In this study, increased doses of the UCNPs were incubated with B16-F10 and A549 cell lines to identify the optimum doses that are able to fluoresce efficiently, while not showing cellular toxic effects, in vitro.

**Figure 4:**
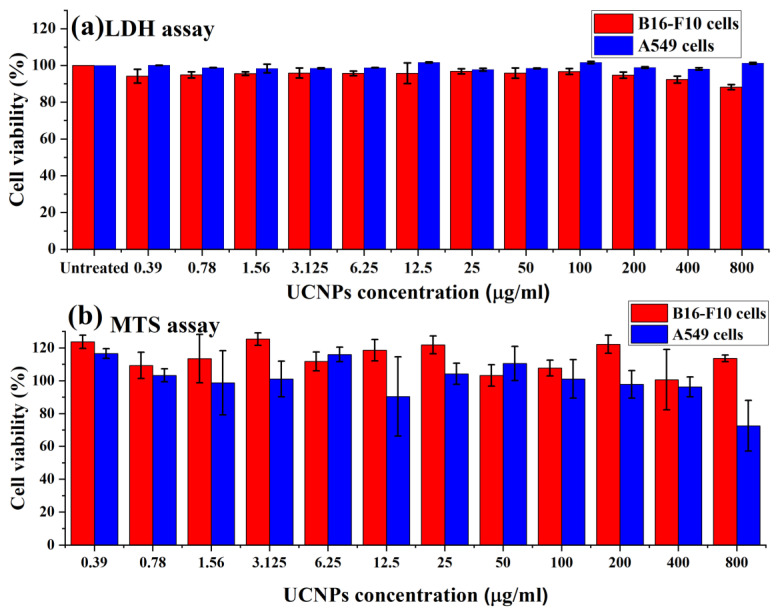
Cell viability of UCNPs after incubation with B16-F10 and A549 cells for 4 h. The data are the result of (**a**) LDH and (**b**) MTS assays, which are expressed as cell viability (%) and presented as the mean ± SD (*n* = 3).

Figure 4a shows the cellular viability of B16-F10 and A549 cells following the treatment with the UCNPs for 4 h, and evaluated using LDH assay. The results exhibited high cell viability (above 80%) for applied doses (from 0.39 to 800 µg/mL).

Figure 4b shows the metabolic activity of B16-F10 and A549 cells in terms of the percentage of cell viability, assessed using MTS assay. The results demonstrated high metabolic activity of both cells and no negative effect of the UCNPs on the cell viability. However, increasing the dose from 400 µg/mL in A549 cells to 800 µg/mL showed a significant decrease in the cell viability from 96% to around 72%.

Following the cells exposure to the UCNPs, the overall results of the cellular viability assays demonstrated no observed change in the viability profile when increasing the concentration of applied nanoparticles, apart from the effect of the highest concentration used in A549 cells (800 µg/mL). The in vitro assessments of the UCNPs exhibited low cytotoxicity effects on B16-F10 and A549 cells, suggesting these fluorescent probe nanoparticles can be applied safely in the biomedical applications.

## 4. Conclusions

We have synthesized small, bright UCNPs that are well dispersed in water, following a simple and robust protected calcination process. The final particles were less than 10 nm in size and well-crystalline with bright up-conversion emission, without optical intermittency (blinking). These properties make them a good alternative to commonly used fluoride-based UCNPs in many biosensing applications. This protected calcination method is a robust and effective strategy to enhance the optical properties of small water-dispersible fluorescent markers.

## Data Availability

The data presented in this study are available on request from the corresponding author.
